# *Neisseria sicca* Vertebral Osteomyelitis: A Case Report and Literature Review

**DOI:** 10.3390/jcm13237241

**Published:** 2024-11-28

**Authors:** Natasha Priya Dyal, Robert Orenstein, Sandhya Rani Nagarakanti

**Affiliations:** Mayo Clinic Arizona, Phoenix, AZ 85054, USA; dyal.natasha@mayo.edu (N.P.D.); orenstein.robert@mayo.edu (R.O.)

**Keywords:** *N. sicca*, vertebral osteomyelitis, infection

## Abstract

**Background**: Culture-negative vertebral osteomyelitis presents a significant diagnostic challenge. *Neisseria sicca* (*N. sicca*) is a typically benign commensal organism of the upper respiratory tract that rarely causes invasive infections, warranting cautious interpretation if isolated in a single positive culture. This case study details a 62-year-old male diagnosed with vertebral osteomyelitis caused by *N. sicca*, examining diagnostic challenges, treatment, and outcomes. **Methods**: We conducted a comprehensive search on MEDLINE using the keywords “*Neisseria sicca*”, “osteomyelitis”, and “diskitis”. An additional search excluding *N. gonorrhea* and *N. meningitidis* was also performed, revealing a total of four cases of *N. sicca* osteomyelitis in the literature. **Results**: A 62-year-old male with a history of hypertension, type 2 diabetes, atrial fibrillation, and previous L5-S1 spinal fusion presented with fever and back pain in May 2023. Initial imaging revealed T8-T9 vertebral osteomyelitis. Despite a six-week course of IV daptomycin, his symptoms worsened, prompting further evaluation. A CT-guided biopsy with comprehensive testing, including histopathology and microbial cultures, initially identified *N. sicca*. Due to its rarity, additional biopsies were conducted, confirming the infection. IV ceftriaxone was initiated, leading to significant pain improvement, and a subsequent MRI showed near resolution. The patient transitioned to oral cefuroxime, with follow-up confirming complete infection resolution by December 2023. **Conclusions**: This case underscores the importance of a structured diagnostic approach in culture-negative vertebral osteomyelitis to differentiate between commensal contamination and true infection. Repeated positive cultures of *N. sicca* from a sterile site confirmed its role as the causative agent. Early identification and targeted antibiotic therapy are critical to improving outcomes in rare cases of *N. sicca* vertebral osteomyelitis.

## 1. Introduction

Culture-negative vertebral osteomyelitis is an increasingly common condition that presents significant therapeutic challenges, particularly in cases where standard microbial diagnostics fail to identify an organism. This often results from factors such as prior antimicrobial therapy, the presence of fastidious organisms, or insufficient tissue sampling [1]. The initial diagnosis is often based on MR imaging, and attempts are often made to establish a microbiologic diagnosis, followed by attempts to achieve microbial confirmation through blood cultures or direct tissue sampling. These samples have traditionally been sent for aerobic, anaerobic, fungal, and mycobacterial cultures. Newer diagnostic modalities such as broad-range PCR and metagenomics are emerging as potential diagnostics but may be associated with erroneous signals, which could impact proper therapy. When diagnostics are negative, clinicians must choose empirical regimens based on host characteristics and the literature. Identifying an oral commensal bacteria from a single disc space aspiration warrants caution.

*Neisseria sicca* is a Gram-negative, oxidase-positive, encapsulated, aerobic diplococcus that is a commensal organism of the human upper respiratory tract. The identification of a single positive culture of *N. sicca* highlights the need for cautious interpretation. It is one of several non-meningococcal, non-gonococcal species of Neisseria, which include *N. lactamic*, *N. cinerea*, *N. mucosa*, *N. flavescens*, *N. subflava*, *N. elongata*, *N. bacilliformis*, *N. oralis*, and *N. macacae*. Unlike its highly pathogenic counterpart, *N. meningitidis* (which commonly has mucoid colonies), *N. sicca* typically produces small, dry, wrinkled colonies and does not reduce nitrates. 

Although *N. sicca* is occasionally associated with invasive infections like meningitis, endocarditis, and pneumonia, it has rarely been implicated as a cause of vertebral osteomyelitis or diskitis. Our literature review identified only four previously documented cases of vertebral osteomyelitis due to *N. sicca.* Here, we present an additional case of *N. sicca*-related osteomyelitis and provide an overview of the existing cases to better understand its role as a potential pathogen in spinal infections.

## 2. Case

**Figure 1:**
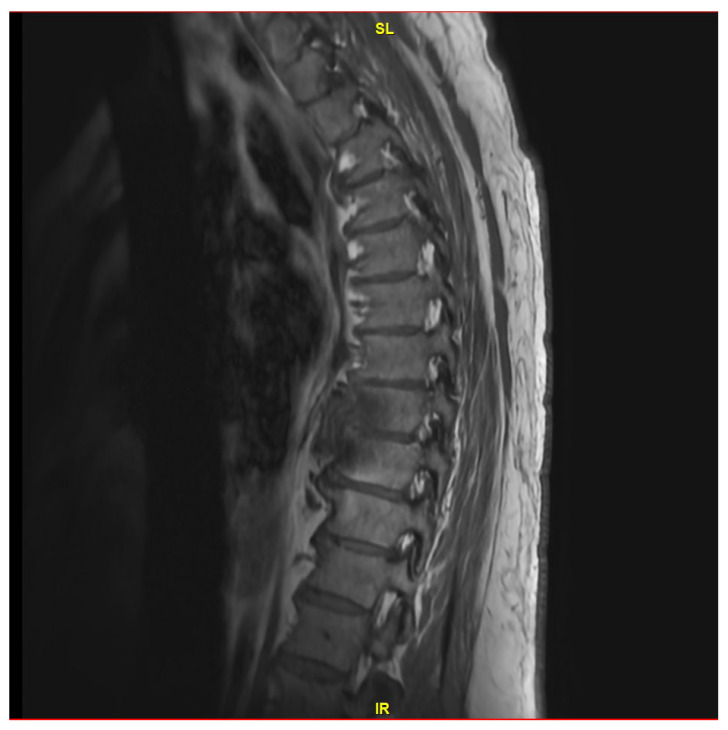
MRI thoracic spine with and without IV contrast revealing small T8 fluid collection and possible diskitis T8-T9.

A 62-year-old male with hypertension, type 2 diabetes, atrial fibrillation (on Eliquis), and an L5-S1 spinal fusion 16 years prior developed fever and back pain on 12 May 2023. He contacted a telehealth provider, who ordered a CT scan of the abdomen and pelvis to evaluate for diverticulitis. This imaging demonstrated no diverticulitis but evidence of T8-T9 vertebral osteomyelitis. He was subsequently hospitalized for 8 days, during which he underwent an MRI of the thoracic and lumbar spine with and without contrast, which showed a T8 vertebral fluid collection and diskitis (Figure 1). C reactive protein was 7.70 mg/L with an erythrocyte sedimentation rate of 16. He underwent a CT-guided biopsy of the impacted vertebra and disc. Stains and cultures for bacteria, mycobacteria, fungi, and histopathologic evaluation performed on these specimens were negative. Because of concern for Staphylococcal (Coagulase negative) infection and convenience of administration, he was discharged on an empiric 6-week course of IV daptomycin 800 mg per day (started on 17 May 2023 and completed on 5 July 2023). His CRP normalized to <0.2 mg/dL at the end of treatment. Despite this treatment, his back pain worsened, for which he was prescribed hydrocodone/acetaminophen (Norco) and a muscle relaxer, metaxalone.

Due to the increasing pain and uncertain diagnosis, his primary care provider checked a serum immunoelectrophoresis to evaluate for possible underlying monoclonal gammopathy. He was found to have two low-level monoclonal IgG kappa chain spikes and was diagnosed with monoclonal gammopathy of undetermined significance.

Two months after his initial presentation, he came to the Mayo Clinic Arizona Division of Infectious Diseases Clinic (on 7 July 2023) for a second opinion. During this visit, it was discovered that he had been experiencing intermittent skin issues, specifically fluid-filled vesicles on the anterior surface of both lower extremities, which became more erythematous while taking antibiotics. He also reported a lesion on his right great toe, which had developed an ulcerated lesion with occasional drainage of black/dark-coloured blood-like discharge without purulence. A plain x-ray of the thoracic spine showed destruction of the anterior/inferior portion of T8; this was followed up with a repeat MRI thoracic spine on 12 July 2023, which now demonstrated progressive findings of osteomyelitis at T8-T9, with progressive destruction extending to T7, without clear abscess. His CRP was elevated at 24.3 mg/L.

He was admitted from 12 July 2023 to 17 July 2023, during which he underwent a CT-guided biopsy. Specimens were sent for histopathological evaluation, bacterial, fungal, and mycobacterial cultures, Coccidioides PCR, and Mycobacteria tuberculosis PCR, and a portion of the specimen was saved for broad-range PCR. The initial sample grew *Neisseria sicca*, but due to the rarity of this causing infection and concerns regarding the adequacy of the sample obtained during CT-guided biopsy, it was decided to obtain additional biopsy samples. The patient underwent a repeat CT-guided needle biopsy from a different site on 17 July 2024. This specimen also demonstrated the growth of *N. sicca*. Given both positive cultures from a sterile site, the infectious disease service recommended treatment with intravenous ceftriaxone 2 g once daily to complete a 6-week course, as this pathogen would not have been susceptible to daptomycin.

**Figure 2:**
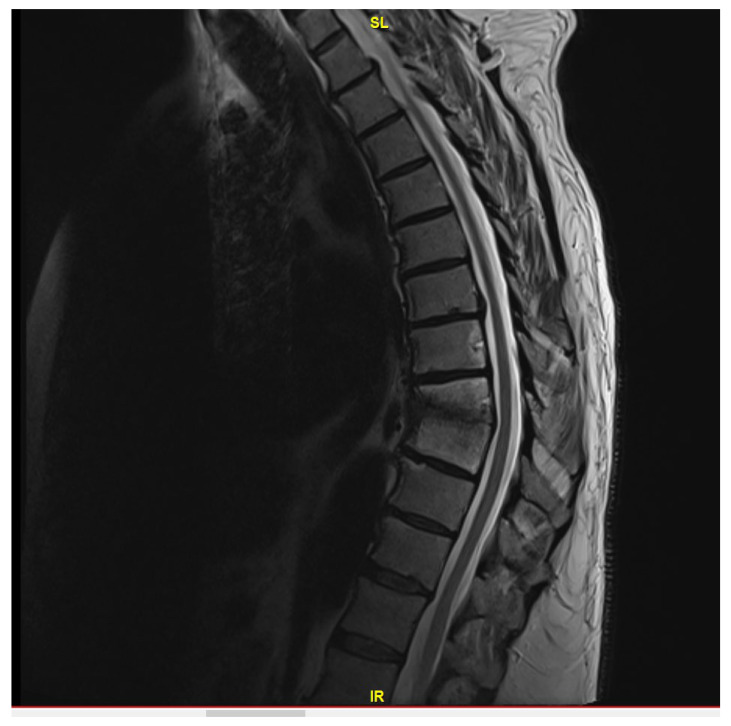
MRI thoracic spine with and without IV contrast revealing redemonstration of T8-T9 diskitis with mild improvement of paraspinal/epidural phlegmon.

He demonstrated progressive improvement in his pain on ceftriaxone without the need for narcotic analgesics. He underwent a third MRI after 6 weeks of treatment (on 29 August 2023), which showed the known changes at T8 and T9, with near resolution of the fluid within the T8-T9 disc (Figure 2). Due to improvement but continued destructive changes and edema, the decision was made to pursue oral therapy after completion of IV ceftriaxone with oral cefuroxime 500 mg q.12 h, which was initiated on 14 September 2023.

**Figure 3:**
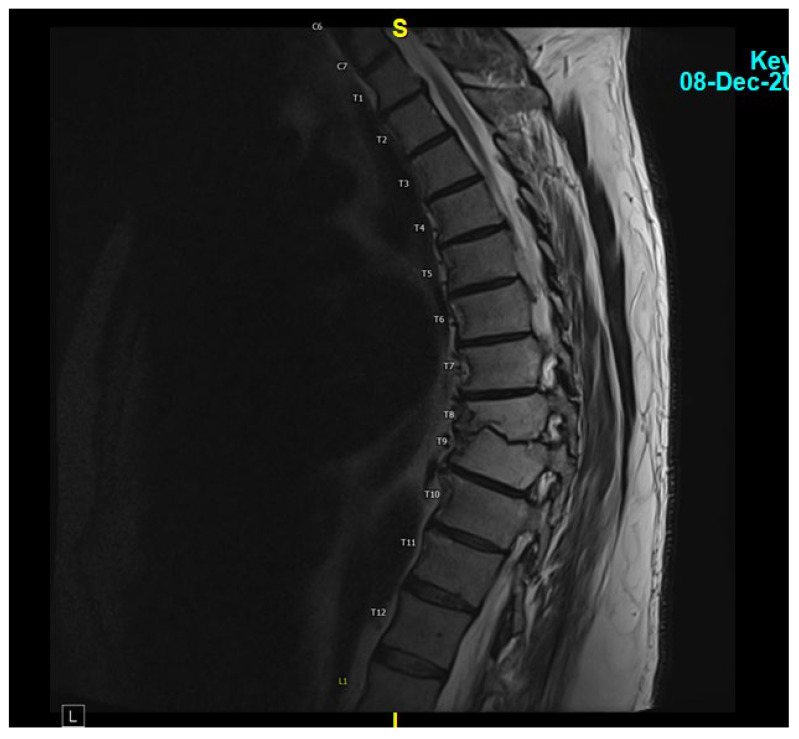
MRI thoracic spine with and without IV contrast showing a completely resolved T8-T9 disc space infection.

He was evaluated by Neurosurgery on 27 September 2023, and they felt the final recommendations for surgical removal of his hardware were not indicated at this time. He followed up with infectious diseases again on 11 December 2023 after undergoing a repeat MRI of the thoracic spine on December 8th, demonstrating complete resolution of the T8-T9 disc space infection (Figure 3). At that time, the patient reported no back pain and was not requiring pain medication. Considering his L5-S1 spinal fusion hardware, he continued his suppressive oral cefuroxime.

At his most recent follow-up, almost one year since the onset of his symptoms, on 11 April 2024, he was well, and through shared decision-making, it was decided to continue his oral suppressive cefuroxime.

## 3. Literature Review

We conducted an extensive search via MEDLINE using the terms “*Neisseria sicca*”, “osteomyelitis”, and “diskitis”. This elucidated one case (described below) [2] All references from this article were reviewed in detail, along with four additional articles from our initial search; however, none of these involved osteomyelitis related to *N. sicca*. An additional arch used filters for infections caused by Neisseria species other than *N. gonorrhea* or *N. meningitidis*. This refined search and a review of the references yielded three additional cases of osteomyelitis involving *N. sicca.*

Below are the details regarding the four previously published cases of the literature regarding *N. sicca* osteomyelitis, which can be summarized in Table 1.

Case Report #1: [2]

A 52-year-old male was transferred to the University of Massachusetts Medical Center with a 6-week history of back pain and fever following a fall from a moving truck. Initially, his back pain had improved, but it recurred with a fever of 39 °C approximately one week before his admission to an outside facility. He was started on empirical cephalothin (2 g IV every 6 h). Initial X-Rays of the lumbosacral spine showed L4-S1 degenerative changes, and subsequent Tc-99 bone scan and myelogram results were normal. Due to persistent fever and back pain after 7 days, he was transferred for further evaluation.

Upon transfer, laboratory workup showed persistent leukocytosis, though not elevated beyond previous levels; the erythrocyte sedimentation rate had increased to 121 mm/h from a normal level noted at the outside facility. Blood cultures taken initially showed no growth after 7 days, leading to the discontinuation of cephalothin. Follow-up X-rays showed degenerative narrowing at the L5-S1 disc space. Seven days after transfer, a Craig needle biopsy of the L5 vertebral body and aspiration of the L5-S1 disc space was performed. Histopathology indicated nonspecific inflammation, but cultures from bone and tissue samples yielded tiny amounts of *N. sicca*. Blood cultures taken at the outside facility also demonstrated growth of *N. sicca* in three out of three samples at three weeks.

A comparison of the antibiograms from the initial blood cultures and the *N. sicca* strain isolated at the University of Massachusetts confirmed identical organisms. The patient was treated with cephalothin (2 g IV every 4 h) and later transitioned to cefamandole, completing a 5-week course. By discharge, he was asymptomatic, and at his 1-year follow-up, he remained symptom-free. Follow-up X-rays showed residual sclerotic densities in the L5 vertebral body without further destruction.

The case report includes minimum inhibitory concentrations (MICs) for the patient’s *N. sicca* isolate: penicillin G, 0.5; ampicillin, 0.8; vancomycin, 3.1; erythromycin, 12.5; trimethoprim/sulfamethoxazole, <0.05/1.0; chloramphenicol, 3.1; rifampin, 0.8; gentamicin, 0.2; cephalothin, 3.1; cefamandole, 0.8; cefotaxime, <0.2; and moxalactam, 0.4.

Case Report #2: [5]

A 37-year-old female with a history of a previously infected sebaceous cyst at the third thoracic vertebra began experiencing pain in the left sciatic nerve distribution approximately one year after the incision and drainage of the cyst. Radiographs of the lumbar spine showed bony proliferation with fusion of the fourth and fifth lumbar vertebrae. Between 1956 and 1962, the patient underwent four surgical interventions. The initial surgery in 1956 was an interlaminar fusion of the L4 through S1 vertebrae. According to the manuscript details, the patient did well for three years before experiencing a recurrence of pain, which led to a second procedure involving surgical aspiration in 1959, followed by additional surgeries in 1961 and 1962.

During the fourth surgery in 1962, intraoperative cultures were obtained due to abnormal findings, including a softened disc space between L2 and L3 and a cavity on the inferior surface of the L2 vertebral body. Cultures from these specimens demonstrated the growth of *N. sicca*. As a result, the patient was treated with erythromycin and penicillin for 17 days. Upon discharge, she showed significant improvement and, at her final outpatient follow-up in August 1963, was noted to be in good condition.

This case highlighted spondyloarthritis as a common cause of lower back pain, often requiring surgical intervention, with infectious spondylitis identified as a rare complication according to available statistics at that time. The exact mechanism of infection in this patient was unknown, though it was hypothesized that it may have been introduced during the third surgical procedure.

Case Report #3: [4]

A 65-year-old otherwise healthy female developed a six-month history of mid-back pain. Initial radiographs of the lumbar spine showed disc height collapse at L4/L5, raising concerns for diskitis. An MRI supported the clinical suspicion of diskitis, prompting a percutaneous biopsy and aspiration of the disc space. Cultures from the disc biopsy tissue demonstrated scant growth of *Neisseria* species, initially reported as a probable contaminant. Due to persistent concern for an underlying infection, the patient underwent an open biopsy. Cultures from the L4/L5 disc tissue again yielded *Neisseria* species, specifically identified as *N. sicca*/*subflava*.

Further analysis of both specimens at the London-based Public Health Laboratory Service confirmed similar biochemical profiles. MIC were determined as follows: penicillin at 0.5 mg/L, amoxicillin at 0.5 mg/L, and cefotaxime at 0.047 mg/L. The patient received a 4-week course of intravenous amoxicillin (500 mg three times daily), followed by a 6-week course of oral amoxicillin (250 mg three times daily). At her 3-month outpatient follow-up after completing antibiotics, she showed complete resolution of her symptoms.

This case underscores the importance of not dismissing unusual pathogens as mere contaminants when clinical suspicion of infection is high. It also highlights the value of withholding antimicrobial treatment in a stable patient while pursuing additional microbiologic testing to confirm the diagnosis.

Case Report #4: [3]

A 60-year-old male with a history of chronic lower back pain and recent radiofrequency ablation and epidural steroid injection presented to a local emergency department with a one-week history of severe, intractable back pain. Initial workup revealed leukocytosis of 15.5, an elevated ESR of 49, and a CRP of 16. MRI of the lumbar spine showed spondylodiscitis, osteomyelitis, paraspinal phlegmon, and extensive complex, septated epidural abscesses with mass effect at L3-L4. He underwent surgical intervention, which included a complete diskectomy at right L3-L4, removal of the infected disc, evacuation of the epidural abscess, and fusion with a titanium cage. Cultures from surgical specimens, including the abscess, disc, and lumbar tissue, demonstrated growth of *Neisseria sicca*/*mucosa*. The patient was treated with a 6-week course of ceftriaxone, resulting in symptom improvement at postoperative follow-up.

This case highlights the importance of considering recent procedures, the presence of prosthetic devices, and the potential for iatrogenic inoculation [6] as risk factors for invasive Neisseria infections.

## 4. Discussion

We have described a case of *N. sicca* thoracic vertebral osteomyelitis, which was effectively treated with ceftriaxone, followed by oral suppressive cefuroxime due to previously placed spinal hardware. *N. sicca* is rarely identified as a cause of human infections. In a 2023 review by Walsh et al., they identified 174 unique articles demonstrative of non-meningococcal, non-gonococcal Neisseria infections: 82 cases of endocarditis, 25 cases of meningitis, 16 of peritonitis, 9 of arthritis, 6 cases of pneumonia, 4 cases each of otitis media and conjunctivitis, 1 case each of urethritis and lymphadenitis, as well as 4 cases of osteomyelitis, and 3 cases of discitis. Analysis of the articles demonstrated that 33% of cases were identified as *N. mucosa*; however, none of the cases specifically isolated *N. sicca* [7].

Given its commensal nature, it follows that *N. sicca* would uncommonly be identified as a pathogen. However, this rarity highlights two potential areas of focus.

The first is microbiologic: *N. sicca* is fastidious, and delays in identifying this organism or misidentifying it as another subspecies of Neisseria have been observed and reported. For example, in a case report by Doern et al., the team at the University of Massachusetts identified small numbers of *N. sicca* colonies from a vertebral biopsy; however, the facility from which the patient had been transferred did not yield *N. sicca* in cultures until 3 weeks after the specimen had been obtained [2]. This underscores the importance of precise microbiologic techniques to ensure proper identification.

*N. sicca*, like other species of the genus, grows optimally on blood and chocolate agar in an atmosphere containing 5% to 10% carbon dioxide at a temperature of 32 °C to 37 °C and pH of 7.0–7.5. While these culture conditions are not particularly difficult to reproduce, they aim to identify some more commonly pathogenic species. Moreover, Neisseria can grossly resemble Moraxella. Therefore, the traditional methods of gross microbiological examination of colonies on agar plates and the performance of in-laboratory tests performed by lab personnel do not have as high or accurate utility as with other, more unique pathogens.

Even within the genus, *N. sicca*, and *N. mucosa* have similar phenotypic characteristics, which highlights the importance of the neck standard step of identifying pathogens after appropriately growing colonies on agar. The most robust mechanism to identify *N. sicca* genomic analysis such as matrix-assisted laser desorption ionization-time-of-flight mass spectrometry (MALDI-TOF). However, there have been reports that MALDI-TOF has misidentified *N. sicca* due to a combination of ANIb and phylogenetic analysis, resulting in a reclassification of strains originally identified as *N. sicca* as *N. subflava* [8,9,10]. This may be because MALDI-TOF identification is performed by creating a score reflective of the reliability of identification of the bacteria to the genus and species level, which is based on available profiles of bacteria within its database. Essentially, MALDI-TOF matches the masses of identified biomarkers, such as 16 RNA gene sequences of unknown organisms, to a library of known bacterial specimens, which are largely obtained from clinical isolates and reference strains. This method of identification presents a problem for low species prevalence bacteria (such as non-meningitidis and nongonococcal Neisseria), as well as those with high levels of diversity and frequent horizontal DNA exchange, which is seen across the genus of Neisseria [9]. This limitation in MALDI-TOF identification of non-meningococcal and nongonococcal species of Neisseria can only be remedied by expanding and reaching reference collections, which is unlikely given the rarity of this bacteria causing a clinically identifiable infectious process. This limitation is studied and presented in detail in regard to the identification of environmental bacteria (such as those in the water supply); conclusions note that for high-diversity bacteria, the lack of entries of reference isolates to a MADI-TOF database can lead to miss identification or unreliable identification [11]. This is precisely why whole-genome sequencing (WGS) remains the most accurate method for identifying non-meningococcal/non-gonococcal species of Neisseria.

WGS can identify bacteria by providing comprehensive genomic information to be used as a reference for laboratory personnel to compare a specific isolate. It therefore bypasses the need to have a profile of a specific bacteria within the database of a single laboratory’s MALDI-TOF apparatus. Moreover, the allowance of detailed phylogenetic analysis by WGS has demonstrated that many strains of *N. sicca* can be clustered into *N. mucosa* or *N. subflava* group [8]. These data are applicable because studies assessing antimicrobial susceptibility more commonly have data on *N. mucosa* and *subflava.*

WGS testing also allows for antimicrobial resistance analysis via examination of genes that may, in turn, inform targeted therapies. Unfortunately, there are no currently established breakpoints for many of the commonly non-pathogenic Neisseria. However current available data suggest that the organism is variably susceptible to cefotaxime, ceftriaxone, and amoxicillin [12,13]. Moreover, depending on the strain, some resistance to penicillin, vancomycin, azithromycin, ciprofloxacin, and aminoglycosides has been observed [12,13].

For example, one study of 96 individuals demonstrated *N. subflava*, *N. mucosa*, *N. oralis*, and other commonly non-pathogenic Neisseria had similar <0.05 MICs as *N. gonorrhea* and *N. meningitidis* for ceftriaxone. Moreover, it demonstrated higher MICs of these non-meningococcal/nongonococcal strains than azithromycin and ciprofloxacin [12]. Unfortunately, *Neisseria sicca* was not among the species studied. Another study’s results showed *N. cinerea*, *N. mucosa,* and *N. elongata*, but not *N. lactamica* displayed higher resistance to chloramphenicol and tetracyclines than the *N. gonorrhoeae* [13]

The decision to transition from intravenous ceftriaxone to oral cefuroxime, in this case, was influenced by cefuroxime’s effective coverage against *Neisseria sicca*. As the patient preferred to avoid surgical removal of spinal hardware and continued to tolerate oral cefuroxime without adverse effects, long-term suppression was chosen as an approach to provide a balance between effective treatment and patient quality of life.

The second highlight is clinical: the degree of suspicion for atypical causes of back pain in cases of radiographically confirmed osteomyelitis/discitis, especially in cases of poor response to pain management, should be high. When organisms are identified via microbiologic methods, it is important to reassess the potential pathogenicity of such organisms, including those commonly identified as contaminants [1]. This is highlighted in the case presented by Roberts et al., detailed above [4]. This is particularly true for organisms identified outside of the usual expected anatomic sites in patients with immunocompromised patients. Neisseria species are well adapted to their primary colonization sites, but their plasticity allows them to colonize alternative anatomic sites [14].

## 5. Conclusions

Culture-negative vertebral osteomyelitis remains a clinical challenge. It is important to perform a structured diagnostic evaluation before proceeding to empirical therapy, which is often prolonged. Failure to make the right empirical choice can lead to worsened outcomes, persistent pain, and complications of therapy. It is rarely an emergency to treat uncomplicated diskitis/osteomyelitis, and obtaining the appropriate samples and diagnostic testing may improve the patient’s outcome. In cases of culture-negative osteomyelitis, histology helps directly visualize the bones, tissue inflammation, and necrosis changes; PCR and next-generation sequencing will identify the bacterial DNA from the tissue. As newer diagnostic modalities using molecular diagnostics become more widely available, it will be important to ensure the specificity of these tests.

In our case, the initial diagnostic test failed to establish a microbiologic diagnosis. The next step should have been additional diagnostic testing before commitment to empirical therapy. If empiric therapy is needed after appropriate testing, then a broader spectrum of coverage should have been provided., which may have targeted the offending organism and saved this patient an additional 6 weeks of parenteral therapy; the few cases of *N. sicca* vertebral osteomyelitis reported highlight the need to pursue early recognition and directed antibiotic therapy. This approach can lead to improved quality of life and symptom resolution for patients, compared with the months-long histories of ongoing pain, multiple surgical interventions, and suboptimal antimicrobial therapy that were previously observed.

## Figures and Tables

**Table 1:** Summary of the 4 prior cases of *N. sicca* vertebral osteomyelitis.

Reference	Title	Journal	Symptoms	Etiology Suspected	Radiology	Micro	Antibiotics
[3]	Commensal Neisseria: A Rare Cause of Osteomyelitis and Discitis	LSUHSC New Orleans Digital Scholar Abstract Submission	Acute chronic back pain for one-week duration.	Iatrogenic inoculation from epidural steroids	Spondylodiscitis, Osteomyelitis, paraspinal phlegmon, and an extensive complex septate epidural abscess with mass effect at L3-L4 on MRI Lumbar spine	*N. Sicca*/*mucosa* by MALDI-TOF	Ceftriaxone for 6 weeks
[4]	Infective discitis with *N. sicca*/*subflava* in a previously healthy adult	Spinal Cord, 2003 Oct; 41(10):590-1.	6-month history of back and bilateral leg pain		Collapse of disc height at L4/L5 with inferior and superior end plate erosion on X-ray and MRI	Disc biopsy culture- scanty growth of Neisseria. Open biopsy 2 weeks later with *Neisseria sicca*/ *subflava*	Intravenous amoxicillin (500 mg TDS) for 4 weeks, then oral amoxicillin (250 mg TDS) for 6 weeks
[2]	*Neisseria sicca* Osteomyelitis	Journal of Clinical Microbiology, Sept. 1982, p. 595–597	Persistent and progressive back pain and fever, 6 weeks after a fall from a moving truck		Degenerative changes at the L5-S1 on X-ray	Needle biopsy of L5 and L5/S1 disc space cx -*N. sicca.*	Cefamandole (2 g every 4 h) for 5 weeks
[5]	Inflammatory Spondylitis	Journal of Neurosurgery, 1965 Apr; 22:393-6	One year of pain at left sciatic nerve distribution. Multiple surgical interventions.		Bony proliferation with fusion of 4th and 5th lumbar vertebrae on X-ray	Culture growth of *N. sicca*	Erythromycin and penicillin for 17 days.

## Data Availability

The original contributions presented in the study are included in the article, further inquiries can be directed to the corresponding authors.
